# The scaffold protein MEK Partner 1 is required for the survival of estrogen receptor positive breast cancer cells

**DOI:** 10.1186/1478-811X-10-18

**Published:** 2012-07-09

**Authors:** Mihaela Marina, Limin Wang, Susan E Conrad

**Affiliations:** 1 Department of Physiology, Michigan State University, East Lansing, MI, 48824, USA; 2 Current address: Department of Radiation Oncology, Winship Cancer Institute, Atlanta, GA, 30322, USA; 3 Department of Microbiology and Molecular Genetics, Michigan State University, East Lansing, MI, 48864, USA

**Keywords:** MEK Partner 1, Estrogen Receptor, Breast Cancer, Cell Survival

## Abstract

MEK Partner 1 (MP1 or MAPKSP1) is a scaffold protein that has been reported to function in multiple signaling pathways, including the ERK, PAK and mTORC pathways. Several of these pathways influence the biology of breast cancer, but MP1’s functional significance in breast cancer cells has not been investigated. In this report, we demonstrate a requirement for MP1 expression in estrogen receptor (ER) positive breast cancer cells. MP1 is widely expressed in both ER-positive and negative breast cancer cell lines, and in non-tumorigenic mammary epithelial cell lines. However, inhibition of its expression using siRNA duplexes resulted in detachment and apoptosis of several ER-positive breast cancer cell lines, but not ER-negative breast cancer cells or non-tumorigenic mammary epithelial cells. Inhibition of MP1 expression in ER-positive MCF-7 cells did not affect ERK activity, but resulted in reduced Akt1 activity and reduced ER expression and activity. Inhibition of ER expression did not result in cell death, suggesting that decreased ER expression is not the cause of cell death. In contrast, pharmacological inhibition of PI3K signaling did induce cell death in MCF-7 cells, and expression of a constitutively active form of Akt1 partially rescued the cell death observed when the MP1 gene was silenced in these cells. Together, these results suggest that MP1 is required for pro-survival signaling from the PI3K/Akt pathway in ER-positive breast cancer cells.

## Background

The small protein MEK Partner 1 (MP1, also known as Map Kinase Scaffold Protein 1 and LAMTOR3) was originally identified as a scaffold protein that potentiates MAPK signaling by binding to MEK1 and ERK1 [1]. MP1 interacts with another small protein p14, and together these two proteins are localized to endomembrane compartments as part of larger signaling complexes. For example, an MP1-p14-MEK1 complex is localized to late endosomes, and this localization is required for EGF-induced ERK1/2 signaling [2-4]. A second MP1-p14-p18 Ragulator complex is required for the recruitment of mTORC1 to the lysosomal surface, and is essential for amino acid-dependent signaling [5]. In addition to these trimeric complexes, MP1 has been reported to bind PAK1 at the plasma membrane, and the MP1-PAK1 interaction is required for MEK phosphorylation by PAK1 in the absence of Raf [6,7]. Thus, MP1 can regulate the function of several intracellular kinases in different subcellular locations.

Both *in vitro* and *in vivo* approaches have been taken to investigate the biological functions of MP1. Transient inhibition of its expression using RNA interference in fibroblasts resulted in decreased Rho activity and delayed cell spreading on fibronectin [7], and similar knockdown experiments in DU145 prostate cancer cells resulted in decreased migration on fibronectin [8]. The effect on migration was independent of MP1’s ability to activate ERK and PAK1, since the levels of phosphorylated ERK and PAK1 were unchanged upon MP1 knockdown. However, MP1 gene silencing in prostate cancer cells was associated with both decreased expression of paxillin and decreased number and turnover of focal adhesions at the migratory edge. Taken together, these data indicate that one function of MP1 in cell culture is related to cell spreading and migration.

Studies performed in conditional p14 knockout mice and in *Drosophila* have addressed the *in vivo* functions of MP1. The endosomal p14-MP1-MEK1 complex is required for cell proliferation in the epidermis during mouse embryogenesis [2]. In *Drosophila*, the MP1/ERK complex regulates cell differentiation during development of the wing, since both down-regulation and overexpression of *dMAPKSP1* led to an ectopic wing vein phenotype [9]. In summary, MP1 is a widely expressed protein that interacts with multiple protein kinases and may impact various cellular processes including proliferation, spreading, migration, and differentiation.

Many of the pathways and processes in which MP1 has been implicated play important roles in cancer biology, including breast cancer. Breast cancer is the most common type of cancer and the second most common cause of death from cancer in women in the United States [10]. A majority of breast tumors express estrogen receptor alpha (ER) and depend on estrogen to grow [11]. There is extensive cross-talk between ER and other cellular signaling pathways, including those in which MP1 functions [12-14]. We therefore hypothesized that MP1 might play an important role in ER-positive breast cancer cells. To test this hypothesis, we analyzed MP1 expression and function in a panel of tumorigenic and non-tumorigenic human mammary epithelial cell lines. Immunoblotting experiments demonstrated that MP1 protein is expressed in both ER-positive and ER-negative breast cancer cell lines, as well as in non-tumorigenic cells. However, the effects of inhibiting MP1 expression by transient transfection with siRNA duplexes differed between different cell types. MP1 gene silencing induced apoptosis of three ER-positive breast cancer cell lines, including one with acquired endocrine resistance. In contrast, no cell death was observed in ER-negative breast cancer or non-tumorigenic cell lines. The apoptosis observed in ER-positive cells was associated with cell detachment, and with decreased ER expression and Akt activity. The cell death phenotype could be partially reversed by overexpressing a constitutively active form of Akt1, suggesting that MP1 plays a novel role in promoting survival of ER-positive breast cancer cells at least in part via the Akt pathway.

## Results

### MP1 protein expression in human mammary epithelial cells

**Figure 1:**
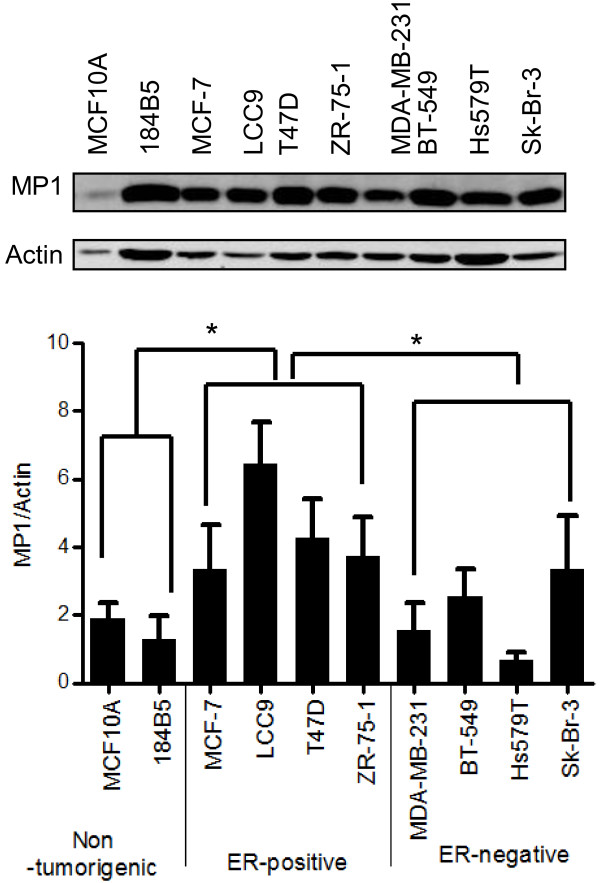
**MP1 expression in breast cancer cell lines.** Human mammary epithelial cell lines were grown in exponential culture and whole-cell lysates were prepared. Top panel: Immunoblot from a representative experiment. Lower panel: Quantitation of MP1/Actin ratios in three independent experiments (mean ± SD, *p < 0.05). For comparing groups of cell lines, an unpaired two-tailed evaluation was done.

MP1 protein expression levels were assessed by immunoblotting in the following human mammary epithelial cell lines: MCF10A and 184B5 (nontumorigenic), MCF-7, MCF-7/LCC9 (LCC9), T47D, and ZR-75-1 (tumorigenic, ER-positive), and MDA-MB-231, BT-549, Hs579T, and Sk-Br-3 (tumorigenic, ER-negative) (Figure 1). MP1 was present in all cell lines, although the level was variable. Actin expression also varied between cell lines, but was consistent between experiments. A comparison between the three categories of cell lines indicated significantly higher levels of MP1 protein in the ER-positive breast cancer cells than in ER-negative breast cancer or non-tumorigenic cell lines.

Since the number of cell lines investigated was limited, we also queried publicly available breast tumor databases for MP1 mRNA expression as described in the methods section. Briefly, eight independent datasets were combined, resulting in a single dataset containing 1459 samples. Batch effects were removed using the BFRM algorithm, and MP1 expression levels were examined. In agreement with the protein results, MP1 mRNA was expressed in both ER positive and negative tumors, but showed a statistically significant elevation in samples that were scored either ER^+^/PR^+^ or ER^+^/PR^-^ by immunohistochemistry (p < 0.001 by t-test for both). Since not all of the samples in the combined dataset were scored for ER and PR, we predicted ER/PR status in the entire dataset using genomic signatures as previously described [15], and compared MP1 levels in ER/PR positive vs. negative tumors. This analysis also indicated that MP1 mRNA is expressed in both groups, but is present at higher levels in ER/PR positive tumors (p < 0.001). One clinical study identified MP1 as a gene associated with a poor prognosis signature in sporadic lymph-node negative breast cancer patients [16], but in our analysis high MP1 expression was not correlated with either time to distant metastasis or disease free survival.

### Inhibition of MP1 expression induces cell death and detachment of ER-positive breast cancer cells

**Figure 2:**
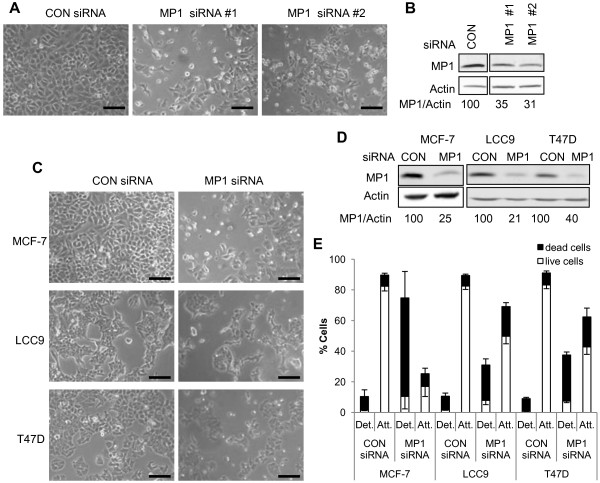
**MP1 expression is required for attachment and survival of ER-positive breast cancer cells.** Cells were transfected with 40 nM control or MP1 siRNAs as described in Materials and Methods. At 48 h cells were photographed, then harvested for counting and extract preparation. (**A**) Photographs of MCF-7 cells transfected with two different MP1 siRNA and control siRNA sequences. Scale bar = 100 μm (**B**) Immunoblots of extracts prepared from cells shown in panel (**A**). Numbers represent the relative MP1/Actin ratios. (**C**) Photographs of MCF-7, LCC9 and T47D cells transfected with MP1 siRNA or control siRNA. Scale bar = 100 μm. (**D**) Immunoblots of extracts prepared from cells shown in panel (**C**). (**E**) Attached and floating cells were collected and analyzed as described in Materials and Methods. The percentage of dead cells (black bars) and live cells (white bars) in each population was determined by trypan blue exclusion assays. Data represents the mean ± SD of three independent experiments. Upper error bars indicate the SD for dead cells and lower error bars represent the SD for live cells.

To study the effect of inhibiting MP1 expression in breast cancer cells, short interfering RNA (siRNA) duplexes were used. Initial experiments were carried out in ER-positive MCF-7 cells. By 48 h post-transfection, cells treated with either of two independent MP1 siRNAs displayed a dramatic phenotype involving cell rounding and detachment (Figure 2A), and by 72 h virtually all cells had detached from the plates (not shown). As shown in Figure 2B, MP1 protein levels were reduced more than 50% with these two MP1 siRNAs relative to control siRNA. To determine if the response to MP1 silencing is a general feature of ER-positive breast cancer cells, two additional ER-positive cell lines were examined: LCC9 and T47D. The LCC9 cell line is an estrogen independent and antiestrogen resistant derivative of MCF-7 cells [17], and T47D is an independently derived ER-positive cell line. MP1 siRNA #1 was used in these experiments. As shown in Figure 2C, both LCC9 and T47D cells exhibited a phenotype similar to MCF-7. To quantitate the effect of MP1 silencing, attached and detached cells were collected at 48 h following siRNA transfection, stained with trypan blue, and counted. As shown in Figure 2E, MCF-7 cells were the most sensitive to MP1 knockdown. More than 70% of MCF-7 cells had detached by 48 h, and the majority of these were dead as determined by trypan blue staining. In contrast, only 10% of cells were detached in the control siRNA transfections. Both LCC9 and T47D cells also showed a significant increase in dead/floating cells upon MP1 silencing, with the average percentage of dead cells at 48 h being 70% for MCF-7, 42% for LCC9 and 49% for T47D (Figure 2E). Thus, MP1 expression appears to be required for the survival of ER-positive breast cancer cell lines, including one with acquired endocrine resistance.

### Inhibition of MP1 expression does not induce death of ER-negative breast cancer cells or non-tumorigenic cells

**Figure 3:**
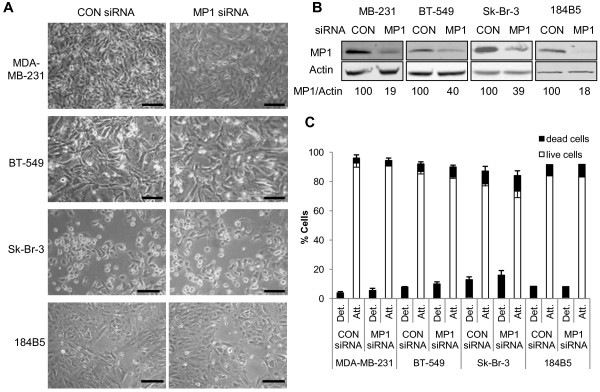
**MP1 expression is not required for attachment or survival of ER-negative mammary epithelial cells.** Cells were transfected with 40 nM control or MP1 siRNAs for all cell lines except 184B5, where 150 nM siRNAs were used, as described in Materials and Methods. At 48 h cells were photographed, then harvested for counting and extract preparation. (**A**) Photographs of transfected MDA-MB-231, BT-549, Sk-Br-3, and 184B5 cells. Scale bar = 100 μm. (**B**) Immunoblots of transfected samples. Numbers represent the relative MP1/Actin ratios. (**C**) Cell counting and trypan blue exclusion assays were carried out as described in the legend to Figure 2. Error bars represent the mean ± SD of three independent experiments for all samples except 184B5 cells. For this cell line the numbers shown represent the average of two independent experiments.

Since MP1 is also expressed in ER-negative breast cancer cells and in non-tumorigenic mammary epithelial cells (Figure 1), the effect of MP1 silencing in representatives of these cell types was also examined. Three ER-negative breast cancer cell lines (MDA-MB-231, BT-549, and Sk-Br-3) and one non-tumorigenic mammary epithelial cell line (184B5) [18] were transfected with either control or MP1 siRNA and examined at 48 h. Although MP1 levels were decreased to the same or greater extent as observed in the ER-positive cell lines, no obvious changes in cell morphology were seen, and cell counting/trypan blue exclusion assays indicated that there was no significant increase in cell detachment or death in compared to control cells (Figure 3). Thus, within this sample, the requirement for MP1 expression for cell attachment and survival was specific to ER-positive breast cancer cells.

### Inhibition of MP1 expression results in apoptosis of MCF-7 cells

**Figure 4:**
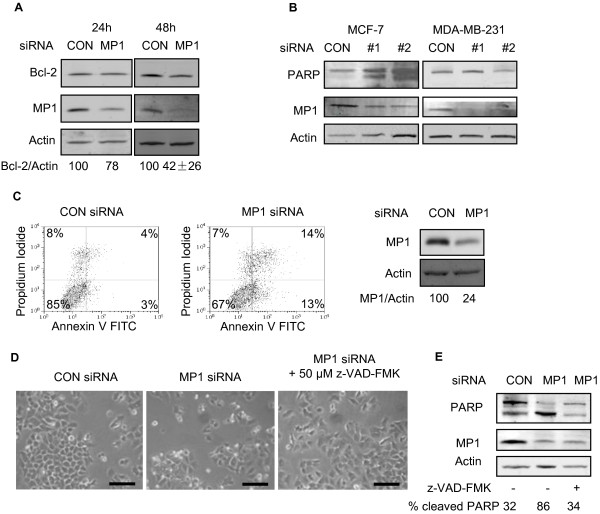
**MP1 silencing induces apoptosis of MCF-7 but not of MDA-MB-231 cells.** MCF-7 or MDA-MB-231 cells were transfected for 24 or 48 h with 30 nM control or MP1 siRNA. (**A**) Immunoblot of Bcl-2 protein levels in extracts of MCF-7 cells. Numbers represent the Bcl-2/Actin ratios expressed as percentage of control samples for a single experiment (24 h)or the average ± SD of 3 independent experiments (48 h, p < 0.05). (**B**) Immunoblot of PARP in extracts of MCF-7 and MDA-MB-231 cells at 48 h. (**C**) Transfected MCF-7 cells were harvested at 48 h, stained with Annexin V and propidium iodide, and analyzed by flow cytometry as described in the methods section. Numbers in the 4 quadrants represent the percentage of cells that are PI-/Annexin- (lower left), PI+/Annexin- (upper left), PI-/Annexin+ (lower right) and PI+/Annexin+ (upper right). The immunoblot of MP1 expression is also shown. (**D**) Photographs of MCF-7 cells transfected with MP1 siRNA in the absence or presence of 50 μM z-VAD-FMK at 48 h. Scale bar = 100 μm. (**E**) Immunoblot and quantification of PARP cleavage at 48 h under the indicated conditions.

To determine if the cell death observed upon MP1 silencing in MCF-7 cells was due to apoptosis, several markers of apoptosis were examined. As shown in Figure 4A, expression of the anti-apoptotic protein Bcl-2 decreased more than two fold in cells treated with MP1 siRNA for 48 h. There was also a small decrease in Bcl-2 expression at 24 h, but knockdown of MP1 expression was not always detected at this time point, so most analyses were done at 48 h. In addition to decreased Bcl-2 expression, cleavage of poly (ADP-ribose) polymerase (PARP), which is a marker of apoptosis, occurred in MP1 siRNA treated MCF-7 cells but not MDA-MB-231 cells (Figure 4B). Consistent with these data, there was a dramatic increase in annexin V-positive apoptotic cells, from 7% to 27%, as a result of MP1 silencing (Figure 4C). To further confirm that MCF-7 cell death was via apoptosis, cells were treated with the pan-caspase inhibitor z-VAD-FMK concurrently with siRNA transfection. As shown in Figures 4D and 4E, this treatment prevented cell rounding/detachment and PARP cleavage in MCF-7 cells.

### MP1 silencing reduces ER protein expression and decreases Akt activity but does not impact ERK expression or activity in MCF-7 cells

**Figure 5:**
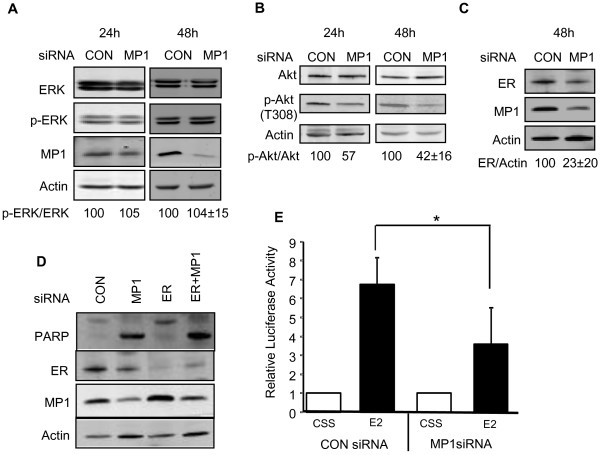
**Effect of MP1 knockdown on cellular signaling pathways.** MCF-7 cells were transfected with 30 nM control or MP1 siRNA for 24 or 48 h. (**A**) Immunoblot of total and phosphor-ERK. The p-ERK/total ERK ratios are expressed relative to the ratio in control samples in a single experiment (24 h) or as the average ± SD of 3 independent experiments (48 h) (p > 0.1). (**B**) Immunoblot of total and phospho-Akt. Ratios of p-Akt/total Akt are expressed as described for panel A. The ratios for the 48 h time point are the average ± SD of 4 independent experiments (p < 0.05). (**C**) Immunoblot of ER at 48 h (n = 3 ± SD, p < 0.05). (**D**) Silencing of MP1 and ER. Cells were transfected with the indicated siRNAs, and extracts were prepared at 48 h. Immunoblots of PARP, ER, MP1, and actin are shown. (**E**) Quantification of luciferase activity in MCF-7 cells. Cells were co-transfected with ERE-tk 109-luc and pβgal-Basic, then with control or MP1 siRNA and treated with medium containing CSS ± E2. The level of luciferase activity in the presence of CSS was set at 1 in each experiment, and the activity in E2 treated samples was expressed relative to this value. The results shown represent the average ± SD of 4 independent experiments. (*p < 0.05).

To identify pathways affected by MP1 silencing, expression of total and phosphorylated ERK and Akt1 were examined (Figure 5). Akt1 is a pro-survival protein with a well-established role in the biology of cancer. ERK is typically associated with proliferation, but may also be involved in regulating cell survival. The level of phospho-ERK was unaffected by MP1 knockdown (Figure 5A), suggesting that a loss of ERK signaling is not responsible for the cell detachment and death observed. In contrast, phospho-Akt1 levels were decreased at both 24 and 48 h after MP1 knockdown (Figure 5B). The effects of MP1 silencing on both ER expression and activity were also examined. As shown in Figures 5C and 5E, both ER levels and ER activity on an ERE-Luc reporter gene decreased 2–3 fold in MP1 siRNA treated cells. To determine if decreased ER levels were responsible for the apoptosis observed, the effects of silencing the ER gene alone or in combination with the MP1 gene were examined. Silencing of ER did not result in apoptosis of MCF-7 cells, indicating that decreased ER expression alone is not the cause of apoptosis in MP1 siRNA treated cells (Figure 5D). In addition silencing ER did not prevent apoptosis induced by MP1 silencing, suggesting that ER expression itself is not required for the apoptotic response.

### Differential requirement for PI3K/Akt pathway for survival of MCF-7 and MDA-MB-231 cells

**Figure 6:**
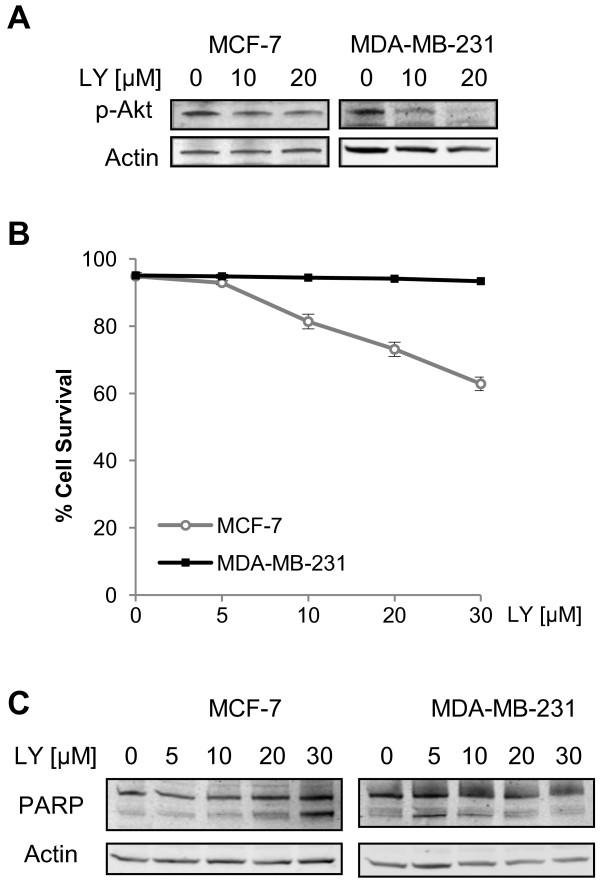
**The PI3K/Akt pathway is required for survival of MCF-7 but not MDA-MB-231 cells.** MCF-7 and MDA-MB-231 cells were treated with various concentrations of LY294002 for 48 h. (**A**) Immunoblot of p-Akt in MCF-7 and MDA-MB-231 cells treated with LY294002. (**B**) Effects of LY294002 treatment on viability as determined by trypan blue exclusion assays (n = 3). (**C**) Immunoblot of PARP cleavage in MCF-7 and MDA-MB-231 cells treated with LY294002.

Inhibition of MP1 expression resulted in cell death in MCF-7 cells, and this was correlated with decreased phosphorylated (active) Akt1 (Figure 5B). In contrast, MDA-MB-231 cells showed no increase in cell death in response to MP1 knockdown. If decreased Akt activity is responsible for the cell death observed after MP1 knockdown in MCF-7 cells, the lack of death in MDA-MB-231 cells could be due to the fact that Akt activity is not dependent on MP1 in MDA-MB-231 cells, or that survival of these cells is not dependent upon active Akt. To test the latter possibility, MCF-7 and MDA-MB-231 cells were treated with various concentrations of the PI3K inhibitor LY294002, and the effects on Akt1 phosphorylation and cell viability were examined. As shown in Figure 6A, a concentration of 20 μM was sufficient to partially inhibit PI3K activity in both cell lines, as indicated by decreased p-Akt1 levels. MCF-7 cell viability declined upon LY294002 treatment (Figure 6B), and this was the result of apoptosis as indicated by increased PARP cleavage (Figure 6C). In contrast, MDA-MB-231 cell viability was unaffected by LY294002 treatment. These data indicate that MCF-7 cells are more dependent on PI3K/Akt1 pro-survival signaling than MDA-MB-231 cells, and are in agreement with previous reports showing a differential requirement for PI3K signaling in these two cell lines [19,20].

### Constitutively active Akt1 partially rescues MP1 siRNA induced apoptosis of MCF-7 cells

**Figure 7:**
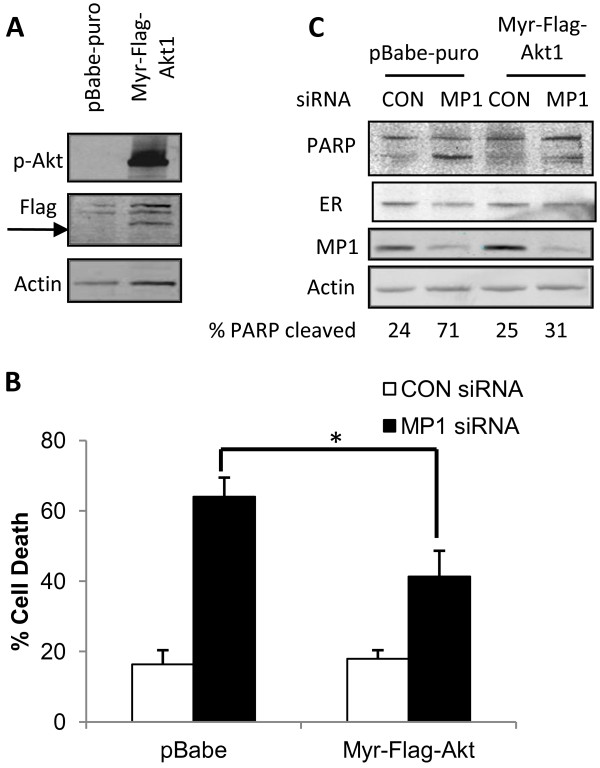
**Constitutively active Akt1 partially rescues MCF-7 cells from apoptosis induced by MP1 gene silencing.** (**A**) Immunoblot of p-Akt and Flag in stable pools of MCF-7 cells infected with control (pBabe-puro) or Myr-Flag-Akt1 expression vector as described in Materials and Methods. (**B**) The stable pools of cells described in (**A**) were transfected with 30 nM control siRNA or MP1 siRNA for 48 h, and cell viability was determined by trypan blue exclusion assay. Bars represent the percentage of trypan blue-positive cells. Error bars represent the mean ± SD for three independent experiments, *p < 0.05. (**C**) Immunoblot of PARP, ER and MP1 in a representative experiment described in (**B**).

MP1 silencing resulted in decreased Akt1 activity in MCF-7 cells, which are highly dependent on pro-survival signals from the PI3K/Akt pathway (Figure 6). To examine whether active Akt1 is sufficient to maintain cell viability in the absence of MP1, we generated MCF-7 cells expressing constitutively active Akt1 (MCF-7/Myr-Flag-Akt1). Phosphorylated-Akt1 (p-Akt) was highly expressed in a pool of MCF-7/Myr-Flag-Akt1 cells compared to a pool of cells containing the control pBabe-puro vector (Figure 7A). These two pools of cells were transfected with MP1 siRNA or control siRNA, and the effects on cell survival were examined. As shown in Figure 7B, 64% of pBabe-puro containing cells were dead in the MP1 siRNA treated sample, but this decreased to 41% in cells expressing constitutively active Akt1. The extent of PARP cleavage in response to MP1 silencing was also decreased in Myr-Flag-Akt1 expressing cells relative to the control cell line (Figure 7C). These experiments were repeated with clonal transfectants containing control vector or Myr-Flag-Akt1 with similar results (Additional file 1: Figure S1). Together, these findings indicate that expression of active Akt1 partially overcomes the requirement for MP1 expression for survival of MCF-7 cells. As shown in Figure 7C, expression of Myr-Flag-Akt1 also rescued the decrease in ER levels that is seen upon MP1silencing. However, this might not be critical to its effect since the results shown in Figure 5 indicate that decreased ER expression does not result in apoptosis.

## Discussion

The results presented here reveal a novel role for the small scaffold protein MP1 in ER-positive breast cancer cells. Although MP1 is expressed in both ER-positive and ER-negative breast cancer cells, its depletion using RNAi-mediated gene silencing leads to detachment and death of several ER-positive cell lines, including one (LCC9) with acquired estrogen independence and antiestrogen resistance. In contrast, MP1 gene silencing had no detectable effect in three ER-negative breast cancer cell lines or a non-tumorigenic mammary epithelial cell line. Although this is a limited sample, MP1 has also been depleted in rat fibroblasts and human prostate cancer cells, and cell detachment or death was not reported in either case [7,8]. Therefore, MP1 expression seems to be required for survival in a subset of cell types, including ER-positive breast cancer cells. The mechanism of cell death that occurs as a result of inhibiting MP1 expression in MCF-7 cells was shown to be apoptosis, as demonstrated by decreased Bcl-2 expression, increased PARP cleavage, Annexin V staining, and rescue of the death phenotype by treatment with the pan-caspase inhibitor z-VAD-FMK.

Several interesting questions are raised by these results. One is what pro-survival pathways are affected by loss of MP1 expression in MCF-7 cells. Depletion of MP1 did not result in decreased ERK activation, indicating that its pro-survival functions are not mediated by the ERK pathway. The lack of an effect on ERK activity was somewhat surprising, since MP1 was originally identified as a scaffold protein that increases ERK signaling [1], but is consistent with results obtained in prostate cancer cells [8]. In contrast, inhibition of MP1 expression resulted in a greater than two fold decrease in Akt phosphorylation. The extent of Akt inhibition may be an underestimate, since by 48 h a majority of cells were dead, and the remaining live cells might represent ones with the lowest extent of MP1 knockdown. Akt plays a known pro-survival function in breast cancer cells, where it relays signals from upstream molecules including integrins, growth factor receptors, PI3K and mTORC1 to downstream molecules such as Bcl-2 and NF-κB [21-26]. In MCF-7 cells, the activity of Akt1 can be modulated by estradiol and IGF [27-29]. The fact that this kinase likely also plays a role in MP1 mediated survival is supported by the fact that expression of a constitutively active Akt1 partially rescued the cell death phenotype observed upon MP1 knockdown. However, the partial effect observed indicates that there are likely to be additional survival pathways involved as well.

Depletion of MP1 in MCF-7 cells also resulted in decreased levels of ERα protein, mRNA (data not shown) and transcriptional activity on ERE-containing promoters. The apoptosis observed is unlikely to be due solely to a loss of ER signaling, since we and others have found that inhibition of ER expression using siRNA does not result in MCF-7 cell death [30]. The fact that LCC9 cells, which are estrogen independent and antiestrogen resistant, die in response to MP1 gene silencing also supports a model in which loss of ER expression is not the sole cause of cell death. Several studies indicate that ER may be implicated in breast cancer cell survival via cross-talk with the PI3K/Akt pathway [31], or by regulating the activity of NF-κB [32,33], Bcl-2 [34,35], or IAP family members [36]. We therefore cannot rule out the possibility that decreased ER expression may contribute in some way to the apoptosis observed in MCF-7 cells.

A second question raised by these results is the molecular basis for the differential requirement for MP1 for survival of ER-positive vs. ER-negative breast cancer cells. One possibility is that activation of pro-survival proteins such as Akt is not dependent on MP1 in ER-negative cells, and a second is that the ER-negative cells are less dependent on these pro-survival signaling pathways. The fact that LY294002 caused a concentration-dependent apoptotic response in MCF-7 cells, but did not affect MDA-MB-231 cells supports the latter hypothesis. This is in agreement with previous reports describing a differential sensitivity to this compound between the two breast cancer cell lines [19,20].

A final question is whether the cell death that we have observed is related to the previously identified roles of MP1 in cell spreading and motility. Since the phenotype involves cell rounding and detachment, inhibition of MP1expression may disrupt cell adhesion signals, which could then trigger cell death. Preliminary PCR array experiments indicated that inhibiting MP1 expression leads to decreased expression of molecules involved in cell adhesion in MCF-7 cells, including several integrins (data not shown). Immunoblotting analysis indicated a small but reproducible decrease in beta 1 integrin protein levels upon MP1 silencing (Additional file 2: Figure S2). Since integrins can initiate pro-survival signaling [37], future experiments will investigate if a loss of integrin expression plays a role in the decreased Akt activation and/or apoptosis observed as a result of MP1 knockdown.

## Conclusions

This is the first report investigating the role of the small scaffold protein MP1 in breast cancer cells. We have demonstrated that inhibiting MP1 protein expression results in apoptosis in ER-positive breast cancer cells, but not ER-negative breast cancer or non-tumorigenic mammary epithelial cells. Furthermore, MP1 gene silencing led to decreased Akt activity in ER-positive MCF-7 cells, these cells are highly dependent upon the Akt pathway for survival, and expression of a constitutively active form of Akt partially rescued cells from apoptosis. We therefore hypothesize that MP1 is required for pro-survival signaling mediated by Akt, and that it may provide a novel target for the treatment of ER-positive breast cancers, including those (such as LCC9) with acquired endocrine resistance.

## Methods

### Cell lines and culture conditions

MCF-7 and LCC9 cells were obtained from the Lombardi Cancer Center. T47D, ZR-75-1, MDA-MB-231, BT-549, and Sk-Br-3 cells were purchased from the American Type Culture Collection. Cells were maintained in Improved Modified Eagle’s Medium (IMEM) containing phenol red (GIBCO-Invitrogen-Applied Biosystems), supplemented with 5% fetal bovine serum (HyClone), and 100 Units/ml Penicillin/100 μg/ml Streptomycin (Invitrogen) and incubated at 37°C with 5% CO_2_.

### siRNA transfections

All siRNA transfection reagents were purchased from Dharmacon-Thermo Scientific. Two independent MP1 siRNA duplexes (ON-TARGETplus), a non-targeting siRNA (ON-TARGETplus siCONTROL) and an ER siRNA (ON-TARGETplus) were used. Cells were plated in six-well plates at 10^5^ to 3 x 10^5^ cells per well in FBS containing medium. After 24 h, cells were transfected with 30–150 nM of either control or MP1 siRNA using DharmaFECT 1 transfection reagent. For MP1 siRNA and ER siRNA cotransfection cells were treated with a 30 nM mix of two duplexes. Cells were harvested after 24 or 48 h, then lysed in CelLytic M lysis buffer (Sigma), supplemented with cocktail tablets of protease (Roche - Complete Mini EDTA-free) and phosphatase inhibitors (Roche – PhosSTOP).

### Determination of cell death

Cell death was assessed at 48 h post transfection using Trypan blue exclusion assays. Briefly, floating cells were collected, centrifuged, and resuspended in PBS, while attached cells were trypsinized, centrifuged, and resuspended in PBS. For each cell suspension, 18 μl were incubated with 2 μl trypan blue for 15 min and both total number and the number of dead cells were counted with a hemacytometer. The remaining harvested cells were processed for protein determination and immunoblotting.

### Luciferase assays

MCF-7 cells were cultured in six-well plates at 3 x 10^5^ cells per well. The following day they were co-transfected with 0.5 μg of ERE2-tk109-luc and 0.06 μg of pβgal-Basic, using Superfect transfection reagent from Qiagen. After 3 h, the medium was changed to transfection mixes containing either control or MP1 siRNA and cells were incubated overnight. The transfection medium was then replaced with phenol red-free IMEM supplemented with 5% charcoal stripped serum (CSS) for 24 h, then cells were stimulated with 10 nM 17β-estradiol (Sigma) for 8 h. Cells were lysed and assayed for luciferase (Promega) and β-galactosidase (Clonetech) activity as suggested by each manufacturer.

### Immunoblotting

Protein concentrations were determined using the Bradford protein assay (Bio-Rad). Total protein (10–20 μg) was subjected to 4-20% Tris–HCl SDS-PAGE (Bio-Rad), transferred to Immobilon-FL polyvinylidene difluoride membranes (Millipore), blocked with Odyssey Blocking Buffer and then incubated with the appropriate primary antibodies. Alexa Fluor 680 anti-goat and anti-rabbit (Invitrogen) and IRDye 800CW anti-mouse (LI-COR) secondary antibodies were used for two-color detection of proteins. Membranes were scanned and analyzed using the LI-COR Odyssey system.

### Antibodies and reagents

The following primary antibodies were used for Western blotting: MP1 (A-19, Santa Cruz), actin (AC-40, SIGMA), estrogen receptor alpha (AB-17, Lab Vision-Thermo Scientific, or F-10, Santa Cruz), PARP (Cell Signaling), p-AKT (T308, Cell Signaling), AKT1 (BDI111, Santa Cruz), ERK (C-16, Santa Cruz), p-ERK (Cell Signaling), Flag M2 (Sigma), or Bcl-2 (BD Biosciences). Pan caspase inhibitor z-VAD-FMK was obtained from BD Biosciences and PI3K inhibitor LY294002 was purchased from Sigma.

### Retroviral infection of MCF-7 cells

pBabe-puro (Addgene plasmid 1764) or pBabe-puro-Myr-Flag-AKT1 (Addgene plasmid 15294, [38]) were transfected into 293GPG packaging cells and retroviral stocks were prepared as previously described [39]. These virus stocks were used to infect MCF-7 cells (1 ml per 10 cm dish), in the presence of polybrene (8 μg/ml), and stable colonies were selected with 0.5 μg/ml puromycin. Both single colonies and pools of 50–100 colonies were selected and propagated. Stable cell lines/pools were routinely maintained in medium supplemented with 0.25 μg/ml puromycin and plated in puromycin-free conditions for siRNA transfections.

### Annexin V staining

Forty eight hours post transfection, floating and attached cells were collected, pooled, washed with PBS, and then prepared for annexin V-FITC and propidium iodide staining according to the manufacturer’s instructions (556547, BD Biosciences). Cells resuspended in 500 μl annexin V binding buffer were analyzed by flow cytometry using a FACSVantage SE system (BD Biosciences) and analyzed using the FlowJo software.

### Gene expression analysis

To examine gene expression across human breast cancer samples, the following 8 breast cancer datasets were downloaded from GEO: GSE2034, GSE3494, GSE6532, GSE4922, GSE11121, GSE7390, GSE2603 and GSE14020. Data was normalized using RMA in the Affymetrix Expression console. The eight datasets were then combined, and batch effects were removed using the BFRM algorithm (Yuwanita and Andrechek, Unpublished). MP1 expression was examined in the resulting combined dataset within the various clinical parameters associated with the datasets, including ER and PR status, time to distant metastasis, and disease free survival. In addition, ER / PR status was predicted using genomic signatures as previously described [15], and MP1 expression levels were compared in hormone receptor positive vs. negative cells. In all cases, p values were calculated using an unpaired two tailed t test.

### Statistical analysis

Data are expressed as the mean ± S.D. Experiments were performed three times unless otherwise indicated. Paired evaluations were made for experimental and control conditions within each set of experiments. For comparing groups of cell lines, an unpaired two-tailed evaluation was done. Significance was determined by Student’s *t* test. Significance level was set at *p <* 0.05.

## Competing interests

The authors declare that they have no competing interests.

## Authors’ contributions

MM designed and conducted experiments, analyzed data and jointly wrote the manuscript. LW designed and conducted apoptosis and flow cytometry experiments, and reviewed the manuscript. SEC conceived of the study, designed experiments, analyzed data and jointly wrote the manuscript. All authors read and approved the final manuscript.

## Supplementary Material

Additional file 1 **Constitutively active Akt1 partially rescues MCF-7 cells from the apoptosis induced by MP1 siRNA. (A)** Immunoblots of total and p-Akt in individual clones of MCF-7 cells infected with control (pBabe-puro) or Myr-Flag-Akt1 expression vector as described in Materials and Methods. The pBabe control-expressing clones did not have detectable levels of p-Akt. The stable clone #1 of control (pBabe-puro) and clone #2 of Myr-Flag-Akt1 expressing cells described in **(A)** were transfected with 30 nM control siRNA or MP1 siRNA for 48 h. **(B)** Immunoblot of MP1. **(C)** Trypan blue exclusion assay. Click here for file

Additional file 2 **Effect of MP1 knockdown on β**_**1**_**integrin protein expression in MCF-7 and MDA-MB-231 cells.** Immunoblot of β_1_ integrin. Anti β_1_ integrin antibody N-20 from Santa Cruz was used (n = 3 ± SD, p > 0.1). Click here for file
