# Supplementary figures and images for: The scaffold protein MEK Partner 1 is required for the survival of estrogen receptor positive breast cancer cells
Source: Cell Commun Signal. 2012 Jul 9;10:18. doi: 10.1186/1478-811X-10-18 (PMC3406937; doi:10.1186/1478-811X-10-18)

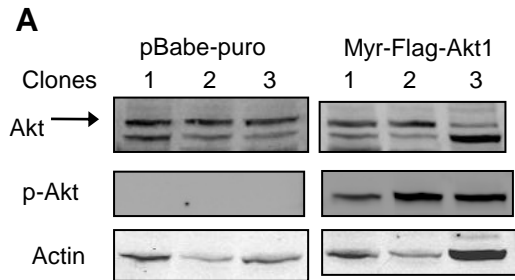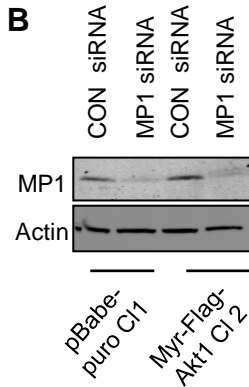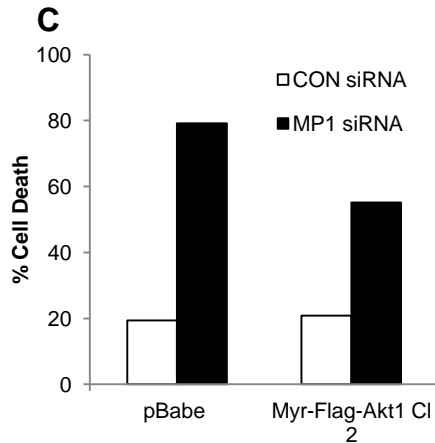

Supplement: Additional file 1 — Constitutively active Akt1 partially rescues MCF-7 cells from the apoptosis induced by MP1 siRNA. (A) Immunoblots of total and p-Akt in individual clones of MCF-7 cells infected with control (pBabe-puro) or Myr-Flag-Akt1 expression vector as described in Materials and Methods. The pBabe control-expressing clones did not have detectable levels of p-Akt. The stable clone #1 of control (pBabe-puro) and clone #2 of Myr-Flag-Akt1 expressing cells described in (A) were transfected with 30 nM control siRNA or MP1 siRNA for 48 h. (B) Immunoblot of MP1. (C) Trypan blue exclusion assay. [file 1478-811X-10-18-S1.pdf]

MCF-7    MDA-MB-231

siRNA    CON   MP1   CON   MP1

IGN $\beta$ 1

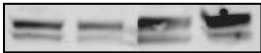

MP1

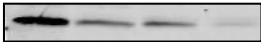

Actin

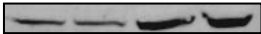

IGN $\beta$ 1/Actin    100   60 $\pm$ 26   100   120 $\pm$ 25

Supplement: Additional file 2 — Effect of MP1 knockdown on β1integrin protein expression in MCF-7 and MDA-MB-231 cells. Immunoblot of β1 integrin. Anti β1 integrin antibody N-20 from Santa Cruz was used (n = 3 ± SD, p > 0.1). [file 1478-811X-10-18-S2.pdf]
